# Immediate visualization of recombination events and chromosome segregation defects in fission yeast meiosis

**DOI:** 10.1007/s00412-019-00691-y

**Published:** 2019-02-09

**Authors:** Dmitriy Li, Marianne Roca, Raif Yuecel, Alexander Lorenz

**Affiliations:** 1 Institute of Medical Sciences (IMS), University of Aberdeen, Foresterhill, Aberdeen, AB25 2ZD UK; 2 Iain Fraser Cytometry Centre (IFCC), University of Aberdeen, Foresterhill, Aberdeen, AB25 2ZD UK; 3 Present Address: Laboratoire de Biologie du Développement de Villefranche-sur-Mer (LBDV), Sorbonne Université, 06230 Villefranche-sur-Mer, France

**Keywords:** *Schizosaccharomyces pombe*, Chromosome segregation, Meiotic recombination, Spore-autonomous promoters, Imaging flow cytometry

## Abstract

*Schizosaccharomyces pombe*, also known as fission yeast, is an established model for studying chromosome biological processes. Over the years, research employing fission yeast has made important contributions to our knowledge about chromosome segregation during meiosis, as well as meiotic recombination and its regulation. Quantification of meiotic recombination frequency is not a straightforward undertaking, either requiring viable progeny for a genetic plating assay, or relying on laborious Southern blot analysis of recombination intermediates. Neither of these methods lends itself to high-throughput screens to identify novel meiotic factors. Here, we establish visual assays novel to *Sz. pombe* for characterizing chromosome segregation and meiotic recombination phenotypes. Genes expressing red, yellow, and/or cyan fluorophores from spore-autonomous promoters have been integrated into the fission yeast genomes, either close to the centromere of chromosome 1 to monitor chromosome segregation, or on the arm of chromosome 3 to form a genetic interval at which recombination frequency can be determined. The visual recombination assay allows straightforward and immediate assessment of the genetic outcome of a single meiosis by epi-fluorescence microscopy without requiring tetrad dissection. We also demonstrate that the recombination frequency analysis can be automatized by utilizing imaging flow cytometry to enable high-throughput screens. These assays have several advantages over traditional methods for analyzing meiotic phenotypes.

**Electronic supplementary material:**

The online version of this article (10.1007/s00412-019-00691-y) contains supplementary material, which is available to authorized users.

## Introduction

Meiosis is a highly conserved process that produces haploid sex cells (gametes) as an integral part of sexual reproduction (Hunter 2015). During meiosis, chromosomes are deliberately broken to initiate homologous (meiotic) recombination that physically connects the equivalent maternal and paternal (homologous) chromosomes; this is absolutely essential for correct chromosome segregation (Petronczki et al. 2003; Lam and Keeney 2015). Only if these connections (chiasmata) are achieved accurately, healthy gametes containing a single chromosome complement will result from the two meiotic cell divisions. In the process, homologous chromosomes are re-shuffled and genes are re-assorted; this provides the genetic diversity that makes individuals unique. Failure to perform meiosis correctly has been shown to cause infertility, miscarriages, and hereditary disorders in mammals (Hassold and Hunt 2001); meiosis is thus fundamental to sexual reproduction.

Meiotic recombination is initiated by Spo11, a topoisomerase VI-like transesterase, creating meiotic double-stranded DNA breaks (DSBs) (Lam and Keeney 2015). These DSBs are subsequently repaired by homology-directed repair mechanisms driven by the RecA-family recombinases Rad51 and Dmc1. Rad51 and its meiosis-specific paralogue Dmc1 are supported by a host of ancillary factors through loading Rad51 and/or Dmc1 onto a processed DSB site and stabilizing them as multimeric nucleoprotein filaments. These ancillary factors include Rad51 paralogues (Gasior et al. 1998; Grishchuk and Kohli 2003; Bleuyard et al. 2005; Sasanuma et al. 2013; Brown and Bishop 2014; Lorenz et al. 2014; Abreu et al. 2018), and factors evolutionarily unrelated to RecA, such as Rad52, Swi5-Sfr1, and Hop2-Mnd1 (Gasior et al. 1998; Chen et al. 2004; Ellermeier et al. 2004; Zierhut et al. 2004; Petukhova et al. 2005; Kerzendorfer et al. 2006; Vignard et al. 2007; Octobre et al. 2008). In *Sz. Pombe*, the Hop2-Mnd1 orthologues are called Meu13-Mcp7, and similar to the situation in other eukaryotes, meiotic recombination is strongly reduced in their absence (Nabeshima et al. 2001; Saito et al. 2004). Homology-directed repair can follow several pathways, and ultimately results in crossover (CO) and non-crossover recombination outcomes (Phadnis et al. 2011; Hunter 2015). Only COs between homologous chromosomes support the formation of chiasmata, which together with sister chromatid cohesion are needed for proper chromosome segregation (Marston 2014). Cohesion is achieved by the cohesin complex which physically entraps the sister chromatids right after their replication during S phase (Nasmyth and Haering 2009). Cohesin holds sister chromatids together until all chromosomes are properly attached to microtubules in metaphase, at which point the kleisin subunit of cohesin is destroyed and anaphase ensues (Nasmyth and Haering 2009; Marston 2014). To reduce the diploid chromosome complement to a haploid one, meiosis consists of two cell divisions following a single round of DNA replication; special modifications to sister chromatid cohesion have to be in place to enable this. During meiosis I, homologous chromosomes are segregated from each other, and cohesins are only removed from the chromosome arms, whereas cohesins at centromeres remain protected for the second meiotic division. During meiosis II, centromeric cohesin protection is removed to allow sister chromatids to be segregated from each other (Petronczki et al. 2003; Marston 2014). A key centromeric protector is the Mei-S332 homolog Shugoshin, Sgo1 (Katis et al. 2004; Kitajima et al. 2004; Marston et al. 2004; Rabitsch et al. 2004), and the absence of Sgo1 and chiasmata, indeed, generates a strong chromosome segregation defect during meiosis (Hirose et al. 2011).

Here, we establish and characterize visual assays to quantify chromosome segregation defects and meiotic recombination frequency which are new to *Sz. pombe*. Visual assays for determining meiotic recombination frequencies were originally established in *Arabidopsis*, and more recently adapted for budding yeast (Francis et al. 2007; Thacker et al. 2011). These visual recombination assays utilize genes encoding red, yellow, and cyan fluorophores driven by gamete-specific promoters, and are integrated at specific loci on a given chromosome to form genetic intervals. The four products (gametes) of a single meiosis will fluoresce in a color corresponding to the fluorophore gene(s) they receive. In *Arabidopsis*, the fluorophores are expressed from the pollen-specific post-meiotic *LAT52* promoter, and various genetic intervals (fluorescent-tagged lines, FTLs) have been generated and adopted widely (e.g., Yelina et al. 2013; Séguéla-Arnaud et al. 2017; Kurzbauer et al. 2018). Also, the budding yeast version of the visual recombination assay starts to enjoy popularity and several recent studies used spore-autonomous fluorophore expression to determine meiotic recombination frequency (e.g., Vincenten et al. 2015; Arter et al. 2018; González-Arranz et al. 2018; Raffoux et al. 2018; Rogers et al. 2018). In yeasts, this kind of setup allows assessment of the frequency of exchange of flanking markers (COs) and has advantages over traditional methods for studying meiotic recombination—such as using nutritional markers (White and Petes 1994; Smith 2009) or Southern blotting of DNA from meiotic yeast cells (Hyppa and Smith 2009; Oh et al. 2009): (I) spores can be assessed regardless of their viability (ability to form a visible yeast colony), (II) the simplicity of this method will allow its use for high-throughput genetic screens, and (III) achieving large sample sizes is straightforward when using imaging flow cytometry. Additionally, this can also be used as a tool for monitoring chromosome segregation defects, when different fluorophore markers are inserted close to a centromere (Thacker et al. 2011; this study).

These visual assays represent a novel, powerful, and easy-to-use experimental tool for fission yeast allowing straightforward analysis of chromosome segregation and homologous recombination defects during meiosis. They also enable the identification and characterization of complex phenotypes (single and double CO formation) in high-throughput screens via imaging flow cytometry.

## Materials and methods

### Molecular and microbiological techniques

Plasmids and details of construction are given in Table S1. DNA-modifying enzymes (high-fidelity DNA polymerase Q5, Taq DNA polymerase, T4 DNA ligase, restriction endonucleases) and the NEBuilder HiFi DNA Assembly Master Mix were obtained from New England BioLabs (NEB), Inc. (Ipswich, MA, USA), and the In-fusion HD Cloning kit from Takara Bio, Inc. (Mountain View, CA, USA). Oligonucleotides (Table S2) were supplied by Sigma-Aldrich Co. (St. Louis, MO, USA). All relevant regions of plasmids were verified by DNA sequencing (Source BioScience plc, Nottingham, UK). Plasmid sequences are available as supporting online material (Lorenz 2018).

*Escherichia coli* was grown in LB and SOC media, when appropriate media contained 100 μg/ml ampicillin (Sambrook and Russell 2000). Competent *E. coli* XL1-blue cells (Agilent Technologies, Santa Clara, CA, USA) were transformed following the protocol provided by the manufacturer.

*Schizosaccharomyces pombe* strains (Table S3) were cultured on yeast extract (YE), and on yeast nitrogen base glutamate (YNG) agar plates containing the required supplements (concentration 250 μg/ml on YE, and 75 μg/ml on YNG). Crosses were performed on malt extract (ME) agar with the required amino acids (concentration 50 μg/ml). Fission yeast transformations were performed using a standard Li-acetate protocol (Brown and Lorenz 2016). Construction of the *hphMX4*-marked *meu13*Δ-*22* strain UoA585 by marker swap from *meu13*Δ::*ura4*^+^ has been described elsewhere (Lorenz 2015); the *meu13*Δ-*43*::*natMX4* strain UoA723 was derived by transforming an appropriate marker swap cassette amplified by PCR (oligonucleotides oUA101 and oUA102, Table S2) from pALo121 into UoA585 (*meu13*Δ-*22*::*hphMX4*) (Lorenz 2015; Brown and Lorenz 2016). Strains carrying the *meu13*Δ-*22*, *meu13*Δ-*43*, *sgo1*Δ, and *rec12*Δ*-169* alleles were derived by crossing from UoA585, UoA723, JG17888, and GP3717, respectively (Davis and Smith 2003; Gregan et al. 2005; Lorenz 2015). A *natMX6*-marked partial deletion of *ade6* (*ade6–3′*Δ::*natMX6*) was created by cloning *natMX6* from pCR2.1-nat as an *Eco*RI-fragment between the *Eco*RI site within the coding sequence and the *Eco*RI site downstream of the STOP codon of *ade6* on plasmid pALo159 (Table S1). The cassette was released from the resulting plasmid (pALo169) by a *Hin*dIII-*Eco*RV restriction digest (Table S1), and transformed into strain ALP729 (Table S3). This generated strain UoA570 (Table S3) carrying a *natMX6*-marked 848 bp deletion at *ade6*, removing 429 bp of coding sequence. All *ade6-3′*Δ::*natMX6* strains have been derived from UoA570 by crossing. Spore-autonomously expressed fluorophore genes were targeted to their intended sites using flanking homologous DNA sequences which were provided via various strategies (Bähler et al. 1998; Matsuyama et al. 2004; Gregan et al. 2006) (Tables S1 and S3).

All sequence details and positional information about *Sz. pombe* genomic loci have been extracted from https://www.pombase.org (Wood et al. 2002).

Spore viability by random spore analysis and meiotic recombination assays have been performed as previously described (Osman et al. 2003; Smith 2009; Sabatinos and Forsburg 2010; Lorenz et al. 2012).

### Microscopy

For microscopy cells from sporulating cultures were suspended in sterile demineralized water, and spotted onto microscopic slides. After placing a cover slip over the cell suspension, cells were immobilized by squashing the slide in a filter paper block, and afterwards the cover slip was sealed with clear nail varnish. Microscopic analysis was done using a Zeiss Axio Imager.M2 (Carl Zeiss AG, Oberkochen, Germany) epi-fluorescence microscope equipped with the appropriate filter sets to detect red, yellow, and cyan fluorescence. A 63× objective (Plan-Apochromat, aperture 1.4) was used for taking black-and-white images with a Zeiss AxioCam MRm CCD camera controlled by AxioVision 40 software v4.8.2.0. For chromosome segregation experiments 9–20 and for recombination assays 20–25 randomly selected fields of view were photographed and evaluated. Images were pseudo-colored and overlaid using Adobe Photoshop CC (Adobe Systems Inc., San José, CA, USA). Images of mature four-spored asci were evaluated manually; data was collected and analyzed in Microsoft Excel 2016 MSO (version 16.0.4738.1000, 32-bit).

### Imaging flow cytometry

The ImageStreamX Mark II (Merck KGaA, Darmstadt, Germany) is an imaging flow cytometer, where an image of each individual cell is acquired as it flows through the cytometer. It measures hundreds of thousands of individual cells in minutes, combining the high-throughput capabilities of conventional flow cytometry with single-cell imaging. The ImageStream measures not only total fluorescence intensities but also the spatial image of the fluorescence plus bright-field and dark-field images of each cell in a population.

For a more extensive overview of data acquisition and analysis in ImageStreamX, see Basiji (2016). Briefly, the INSPIRE acquisition software generates raw image data (.rif file) without compensation which can then be directly loaded into IDEAS for further analysis. Using the IDEAS software, the .rif files will then be converted into compensated image files (.cif) by applying the compensation matrix (.ctm) generated from single fluorescence controls during the acquisition. The file resulting from analysis is stored as .daf (data analysis file), which is used for plotting features derived from the bright-field, dark-field, and fluorescence single cell images in the form of histograms or bivariate scatter plots. Subpopulations are generated using these plots and saved as analysis template for further datasets.

For imaging flow cytometry, cellular material containing asci was suspended in 1× PBS, pH 7.5 (8 g/l NaCl, 2 g/l KCl, 1.15 g/l Na_2_HPO_4_·7H_2_O, 2 g/l anhydrous KH_2_PO_4_), harvested by centrifugation (6000×g, 30 s), and re-suspended in 1× PBS, pH 7.5. Data was acquired on the ImageStreamX Mark II using INSPIRE acquisition software (Merck kGaA). Cellular parameters were measured in Channel 1 (Brightfield, BF), Channel 2 (GFP*, a yellow-shifted version of green fluorescent protein, using a 485 nm laser), Channel 4 (RFP, red fluorescent protein, 561 nm), Channel 7 (CFP, cyan fluorescent protein, 405 nm), and Channel 12 (side scatter, 785 nm) with magnification set to 60×. Briefly, objects of interest (asci) with a BF “area” of 50 to 200 μm^2^ and an “aspect ratio” (ratio of minor axis to major axis) lower than 0.5 (“doublet area”) were selected. Focused cells were identified by a “gradient RMS” feature value of 50 or higher. A typical file contained about 25,000 focused yeast cells.

Data evaluation for identification of asci and spore phenotyping were performed using IDEAS software (version 6.2; Merck). A focused population of asci were identified within the “doublet area” and based on the features “Modulation” for fluorescent channels (the Modulation texture feature measures the intensity range of an image, normalized between 0 and 1) and “Intensity” for side scatter (SSC) using the custom masks “Morphology” and “Object(right),” respectively. Further refinement was performed each on RFP, GFP*, and CFP fluorescence via “Intensity.” Following analysis of the merged triple fluorescent population using “Length” and “Elongatedness” (ratio of the height over width of the object’s bounding mask) features (custom BF mask “AdaptiveErode, M01, Ch01, 75”) resulted in identification of asci of interest. Finally, spore phenotype analysis was conducted by evaluating the fluorescent area using custom masks for each fluorescent intensity (GFP* intensity 200–4095, RFP intensity 75–4095, and CFP intensity 150–4095) and by applying Boolean algebra to identify particular combinations of fluorescent colors. Asci with a mask area larger than 3 μm^2^ were considered positive for a particular spore phenotype.

## Results and discussion

### Identifying spore-autonomous promoters in *Schizosaccharomyces pombe*

To test whether a particular upstream regulatory sequence is a spore-autonomous promoter (Thacker et al. 2011), we cloned a 700–931-bp region upstream of the start codon of the *Sz. pombe eis1*, *pil2*, *eng2*, *agn2*, and *mde10* genes in front of a cyan (*mCerulean*) or red (*tdTomato*) fluorophore gene inserted in pDUAL, a vector restoring *leu1*^+^ by integrating at the *leu1–32* mutant locus (Matsuyama et al. 2004). Fluorophore genes were terminated by *Saccharomyces* spp. *PGK1* downstream regulatory sequence: *T*_*PGK1*_ from *S. bayanus* for *mCerulean*, and *T*_*PGK1*_ from *S. kudriavzevii* for *tdTomato* (Thacker et al. 2011). The candidate promoters were selected on the basis of its corresponding gene being upregulated during late meiosis or sporulation (Mata et al. 2002): *eng2*, *agn2*, and *mde10* code for proteins involved in spore wall formation, *eis1* encodes an eisosome assembly protein, and *pil2* a component of the eisosome. The promoter of *S. cerevisiae YKL050c* has previously been shown to support spore-autonomous expression of fluorophores in budding yeast (Thacker et al. 2011); *Sz. pombe eis1* is the single homolog of the *S. cerevisiae* paralogue pair *EIS1* and *YKL050c*. The resulting plasmids (pALo139, pALo140, pALo141, pALo142, pALo175; Table S1) were digested with *Apa*I to release the *leu1*^+^ integration cassettes containing the constructs; these were transformed into *h*^*+*^ and *h*^*−*^ fission yeast strains (ALP729 and FO652) carrying the *leu1–32* mutation. Two *leu1*^+^ strains of different mating types carrying differently colored fluorophore constructs were crossed to each other, and presence or absence of spore-specific fluorescence was recorded on an epi-fluorescence microscope. *P*_*eng2*_, *P*_*agn2*_, and *P*_*mde10*_ failed to produce fluorescence levels visible under the microscope (data not shown). *P*_*eis1*_ and *P*_*pil2*_ were strong spore-autonomous promoters yielding clear red or cyan fluorescence in spores of mature asci (data not shown).

**Fig. 1:**
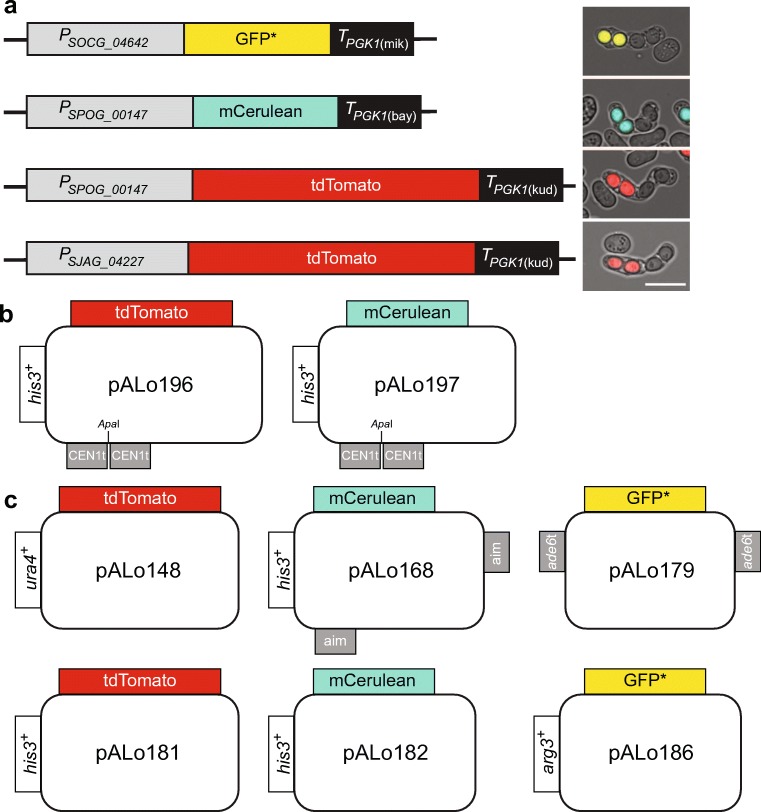
Spore-autonomous expression of fluorophores. **a** Schematic and examples of main constructs, *P*_*SOCG_04642*_-GFP*-T_*PGK1(mik)*_ from strain UoA694, *P*_*SPOG_00147*_*-mCerulean-T*_*PGK1(bay)*_ from strain UoA727, *P*_*SPOG_00147*_*-tdTomato-T*_*PGK1(kud)*_ from strain UoA726, and *P*_*SJAG_04227*_*-tdTomato-T*_*PGK1(kud)*_ from strain UoA694; scale bar in example images represents 10 μm. **b** Plasmid maps of *CEN1*-targeting (CEN1t) constructs using the *Sz. octosporus SPOG_00147* (*pil2*) promoter to drive RFP (tdTomato) and CFP (mCerulean) expression. **c** Plasmid maps of constructs usable for generating genetic intervals (see main text for details); RFP is driven by the *Sz. japonicus SJAG_04227* (*eis1*) promoter in pALo148 and by *Sz. octosporus SPOG_00147* (*pil2*) promoter in pALo181, CFP by the *Sz. octosporus SPOG_00147* (*pil2*) promoter in pALo168 & pALo182, and the yellow-shifted GFP* by the *Sz. cryophilus SOCG_04642* (*pil2*) promoter in pALo179 & pALo186

To avoid ectopic recombination events between the *P*_*eis1*_ and *P*_*pil2*_ constructs and the upstream regions of endogenous *eis1* and *pil2*, we decided to follow a similar strategy as Keeney and co-workers (Thacker et al. 2011), and investigated whether *P*_*eis1*_ and *P*_*pil2*_ from *Schizosaccharomyces* species other than *Sz. pombe* can be used as spore-autonomous promoters in *Sz. pombe*. Indeed, the upstream sequences of the *Sz. japonicus eis1* and *pil2* homologs *SJAG_04227* and *SJAG_02707*_*,*_ as well as the regions upstream of *Sz. cryophilus* and *Sz. octosporus pil2* homologs *SPOG_00147* and *SOCG_04642*, cloned in front of fluorophores produced strong fluorescence in spores of *Sz. pombe* asci (Fig. 1a). *P*_*SJAG_04227*_, *P*_*SPOG_00147*_, and *P*_*SOCG_04642*_ were selected to drive tdTomato (red fluorescence protein, from now called RFP), GFP* (yellow-shifted green fluorescence protein, terminated by *T*_*PGK1*_ from *S. mikatae*) (Griesbeck et al. 2001; Thacker et al. 2011), and mCerulean (cyan fluorescence protein, from now on called CFP) expression in all experimental constructs (Fig. 1).

### Monitoring meiosis chromosome segregation defects

**Fig. 2:**
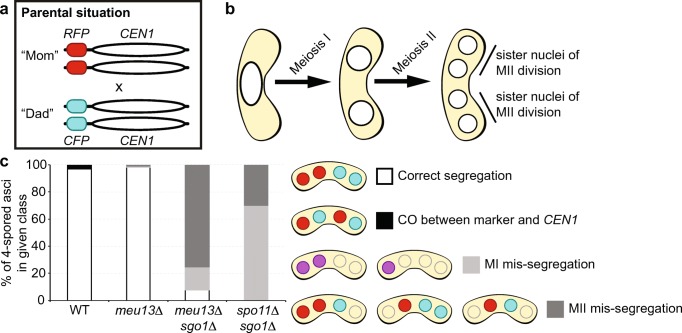
Chromosome segregation assay using spore-autonomous expression of fluorophores. **a** Schematic of assay, RFP and CFP are expressed from the *Sz. octosporus SPOG_00147* (*pil2*) promoter integrated at position 3,751,911 on chromosome 1 downstream of the *per1* (*SPAP7G5.06*) locus close to its centromere (*CEN1*). **b** Meiotic nuclear divisions generate an ordered tetrad with sister nuclei from meiosis II (MII) ending up next to one another. **c** Chromosome segregation phenotypes in four-spored wild-type (WT; UoA726 × UoA727, *n* = 274), *meu13*Δ (UoA752 × UoA755, *n* = 101), *meu13*Δ *sgo1*Δ (UoA756 × UoA759, *n* = 53), and *spo11*Δ *sgo1*Δ (UoA760 × UoA763, *n* = 20 asci) asci. A low frequency of crossover (CO) events (3.3%) between the fluorophore genes and *CEN1* has been observed in WT

Markers inserted next to the centromere can be used to monitor meiotic chromosome segregation defects. Previously, this has been exploited in genetic screens by introducing bacterial operator repeats (*lacO* or *tetO*) close to centromeres in budding and fission yeast, to identify chromosome segregation mutants via the distribution of LacI- or TetR-GFP fusions binding to their respective operators, thus becoming visible as small foci (Straight et al. 1996; Michaelis et al. 1997; Nabeshima et al. 1998; Katis et al. 2004; Rabitsch et al. 2004; Gregan et al. 2005). Introducing spore-autonomously expressed fluorophore markers with different colors at the centromere (Figs. 1b and 2a) has the advantages of (I) enabling distinction of meiosis I and meiosis II segregation defects in a single assay (Fig. 2) rather than requiring homozygous and heterozygous setups of *lacO* or *tetO* repeats integrated close to a centromere, and (II) likely not interfering with chromosome behavior as strongly as *lacO* or *tetO* repeats (Fuchs et al. 2002; Sofueva et al. 2011). Fission yeast asci are ordered; due to the physical constraints of the zygotic cell size and shape, microtubular spindles can orientate only along the longitudinal axis of the zygote, which means that the neighboring nuclei/spores in one half of the zygote are the sister products generated in meiosis II (Fig. 2b). This makes the evaluation of chromosome mis-segregation a comparatively straightforward undertaking in *Sz. pombe*.

The integration of spore-autonomously expressed fluorophore cassettes at the centromere of chromsome 1 (*CEN1*) was enabled by sequences homologous to a genomic region downstream of the *per1* (*SPAP7G5.06*) locus (position 3,751,911 on chromosome 1), similar to a strategy developed for high-throughput gene deletion in fission yeast (Gregan et al. 2006). The *CEN1*-targeting plasmids carrying a *his3*^+^ selection marker and the spore-autonomously expressed fluorophore *P*_*SPOG_00147*_*-tdTomato* (pALo196) or *P*_*SPOG_00147*_-*mCerulean* (pALo197) were linearized by an *Apa*I restriction digest and transformed into yeast strains ALP729 or FO652 (Tables S1 and S3). All strains carrying *CEN1*::*P*_*SPOG_00147*_-*tdTomato* were generated by crossing from UoA726 (ALP729 transformed with *Apa*I-digested pALo196), and all strains carrying *CEN1*::*P*_*SPOG_00147*_-*mCerulean* were derived from UoA727 (FO652 transformed with *Apa*I-digested pALo197) by crossing.

We tested the functionality of our assay carrying fluorophore genes under the control of the spore-autonomous *SPOG_00147*-promoter integrated close to *CEN1* (Fig. 2a) with a set of mutants defective in meiotic recombination (*meu13*, *spo11*) and/or kinetochore function (*sgo1*) (Keeney et al. 1997; Nabeshima et al. 2001; Sharif et al. 2002; Rabitsch et al. 2004). For this, we only evaluated four-spored asci, and ignored asci with spore counts of 1, 2, or 3, to exclude incidences of clear nuclear division failures in meiosis I or II. As expected, in wild-type and *meu13*Δ crosses, chromosome 1 is correctly segregated, in almost all cases (Fig. 2c). We did observe a low frequency (3.3%) of CO recombination between the fluorophore marker and the physical centromere in wild type, leading to red–cyan pairs of sister nuclei, rather than red–red and cyan–cyan pairs (Fig. 2c). In *meu13*Δ, which strongly reduces meiotic recombination (Nabeshima et al. 2001), no COs were observed, but two incidences of chromosome mis-segregation could be recorded (Fig. 2c). As an obvious example for meiotic chromosome mis-segregation, we employed double mutants of *sgo1*Δ with *meu13*Δ or *spo11*Δ. A *sgo1*Δ single mutant does not produce a strong mis-segregation phenotype (Rabitsch et al. 2004), but in combination with the absence of recombination factors, a meiotic non-disjunction phenotype can be observed (Hirose et al. 2011). Indeed, massive chromosome segregation defects are obvious in asci of *meu13*Δ *sgo1*Δ and *spo11*Δ *sgo1*Δ double mutants (Fig. 2c). In *spo11*Δ *sgo1*Δ, the percentage meiotic non-disjunction is slightly higher than in *meu13*Δ *sgo1*Δ, and there are also more meiosis I chromosome mis-segregation events in *spo11*Δ *sgo1*Δ. In *meu13*Δ, chromosome segregation can presumably be supported to some degree, because a small number of chiasmata is still being produced, whereas in *spo11*Δ meiotic DSB formation is completely abrogated and thus no chiasmata are formed.

### Creating a genetic interval with fluorophore markers to assess meiotic recombination frequency

**Fig. 3:**
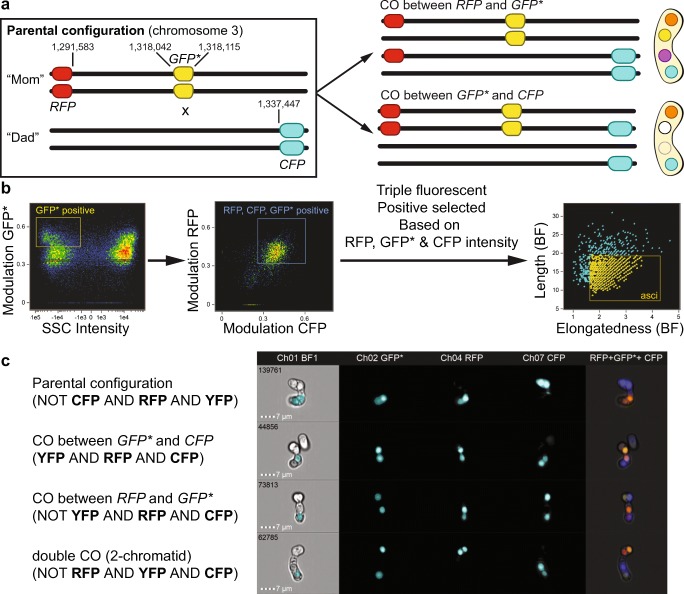
Genetic interval constructed with spore-autonomously expressed fluorescent markers can be analyzed by imaging flow cytometry. **a** Schematic of the genetic interval constructed: *RFP* expressed from the *Sz. japonicus SJAG_04227* (*eis1*) promoter together with a *ura4*^+^ marker is integrated on chromosome 3 at position 1,291,583, the same site as *ura4*^+^-*aim2* (Osman et al. 2003); *CFP* expressed from the *Sz. octosporus SPOG_00147* (*pil2*) promoter together with a *his3*^+^ marker is integrated on chromosome 3 at position 1,337,447, the same site as *his3*^+^-*aim* (Osman et al. 2003); GFP* expression driven from the *Sz. cryophilus SOCG_04642* (*pil2*) promoter, the construct is integrated between positions 1,318,042 and 1,318,115 on chromosome 3 (downstream of *ade6* at its endogenous locus). Only outcomes of single crossovers (COs) between the three markers are shown; double COs are rare (see Figs. S2 and S3 for double COs observed in this kind of assay). Please note that order of spore colors is not fixed, but can rotate perpendicular to the meiotic spindle axis. **b** Outline of the workflow to identify asci based on particular cellular features on the Amnis ImageStreamX Mark II. Modulation measures the intensity range of an image normalized between 0 and 1 by calculating Modulation = (Max Pixel − Min Pixel)/(Max Pixel + Min Pixel). **c** Examples of ascus phenotypes from a cross of wild-type strains (UoA694 × UoA676) as shown in **a**; Boolean algebra mask equations used to discriminate between the different ascus types as presented in Ch01 BF1

To explore whether fluorophore markers inserted at defined genomic sites on a single chromosome to create a genetic interval that can be used to determine meiotic recombination frequencies, we transformed constructs integrating on chromosome 3 forming a genetic interval of ~ 45 kb around the *ade6* locus (Fig. 3a) (Osman et al. 2003; Lorenz et al. 2012). To target the *ura4*^+^*-*marked *P*_*SJAG_04227*_*-tdTomato* construct to the same locus as *ura4*^+^-*aim2* on chromosome 3 (at position 1,291,583, ~26.5 kb upstream of *ade6*), a *ura4*^+^*-P*_*SJAG_04227*_*-tdTomato*-*T*_*PGK1(Skud)*_ cassette was amplified by PCR from pALo148 adding ~80 bp of homologous flanking sequences (oligonucleotides oUA113 and oUA114, Table S2) (Bähler et al. 1998), and transformed into strain FO652. All strains harboring *ura4*^+^*-aim2-P*_*SJAG_04227*_*-tdTomato* have been derived from the resulting transformant, UoA523 (Table S3), by crossing. A similar approach failed to deliver the *his3*^+^*-P*_*SPOG_00147*_*-mCerulean* to the same site as *his3*^+^-*aim* on chromosome 3 (at position 1,337,447, ~ 19.5 kb downstream of *ade6*). Therefore, we cloned larger homologous flanking sequence up- and downstream of *his3*^+^*-P*_*SPOG_00147*_*-mCerulean*-*T*_*PGK1(Sbay)*_ into the *Not*I sites of pALo182 (Table S1). The backbone and insert (*his3*^+^*-P*_*SPOG_00147*_*-mCerulean*-*T*_*PGK1(Sbay)*_) of pALo182 after a *Not*I digest were merged with a 436-bp upstream (oligonucleotides oUA189 and oUA190) and a 646-bp downstream flanking sequence (oligonucleotides oUA191 and oUA192, Table S2) amplified by PCR from *Sz. pombe* genomic DNA (strain MCW1196, Table S3) in a single NEBuilder assembly reaction (in the process, the *Not*I sites flanking the whole construct were replaced by *Sma*I sites). The whole cassette was excised from the resulting plasmid (pALo168, Table S1) by *Sma*I digestion and transformed into strain ALP729. This generated strain UoA676 (Table S3), from which all strains carrying *his3*^+^*-aim-P*_*SPOG_00147*_*-mCerulean* were derived by crossing. Finally, the yellow spore marker (*P*_*SOCG_04642*_-GFP*-*T*_*PGK1(Smik)*_) on pALo179 was generated by an NEBuilder assembly of *P*_*SOCG_04642*_ (PCR on genomic DNA of *Sz. octosporus* yFS286, oligonucleotides oUA201 and oUA202) and GFP*-*T*_*PGK1(Smik)*_ (PCR on pSK726, oligonucleotides oUA204 and oUA138) between the *ade6*-targeting sequences on pALo159 (linearized by a *Bam*HI and *Bgl*II digest) (Tables S1 and S2). The *ade6*^+^::*P*_*SOCG_04642*_-GFP* strain UoA666 (Table S3) was created by transforming the *ade6*-targeting cassette from pALo179 (amplified by PCR, oligonucleotides oUA142 and oUA143; Table S2) into the *ade6-3′*Δ::*natMX6* strain UoA570. This transformation restored *ade6-3′*Δ to *ade6*^+^ and removed the *natMX6* cassette, all *ade6*^+^::*P*_*SOCG_04642*_-GFP* strains were derived from UoA666 by crossing.

As this assay visualizes recombination events, we evaluated it using standard epi-fluorescence microscopy, and also tested whether single-cell imaging flow cytometry (Basiji 2016) could be exploited to perform high-throughput screens with the spore-autonomously expressed fluorophore recombination assay. We established a workflow on the Amnis ImageStreamX Mark II imaging flow cytometer to select for mature asci displaying fluorescence from a mixed population of cells in a standard cross (mature fluorescing asci, immature non-fluorescing asci, zygotes, vegetative cells), and subsequently applied customizations in the software to identify spore color phenotypes unique for the recombination outcomes we expected to occur in this assay (Fig. 3b, c).

The important first steps are identifying focused cells by using a measure of the “gradient RMS” feature of the bright-field image to define the focus quality. Single and multiple cells were determined by plotting the cell mask area versus cell mask aspect ratio, whereby the asci were located in the doublet area. Once focused subspecies are identified via gating, they were used as the starting point to analyze recombination products by determining the spore phenotype, which is only feasible by utilizing the fluorescent markers GFP*, RFP, and CFP.

For this purpose, mainly the “Modulation (texture)” feature was applied to objectively discriminate the bright fluorescence pattern of GFP*, RFP, and CFP associated asci. We first gated GFP*-positive objects on the basis of the appropriate “Modulation (texture)” against darkfield using the side scatter (SSC) parameter in a bivariate plot. In the next stage, the gated GFP* population was subanalyzed for the modulation of RFP- and CFP-containing spores (Fig. 3b).

Employing the ability of the IDEAS software for creating Boolean logic, masks with good determination of spore borders in each fluorescent channel were selected, and these advanced combined masks determined spores with particular fluorescent phenotypes (Fig. 3c). For example, spores with all three fluorescent proteins are only possible, if recombination happened between GFP* and CFP, whereas spores containing RFP and CFP are only possible, if recombination happened between RFP and GFP*. Finally, asci were quantified within the triple merged combined fluorescent populations by using the newly created shape features “Length” versus “Elongatedness” in brightfield. Thereby, asci with recombination products were identified within a “Length” < 20 and an “Elongatedness” > 2 (Fig. 3).

If a particular experimental setup requires a distinction between four-spored asci and asci with irregular spore numbers (1, 2, 3, > 4), masks using information from the brightfield channel can be programmed to accommodate this.

**Fig. 4:**
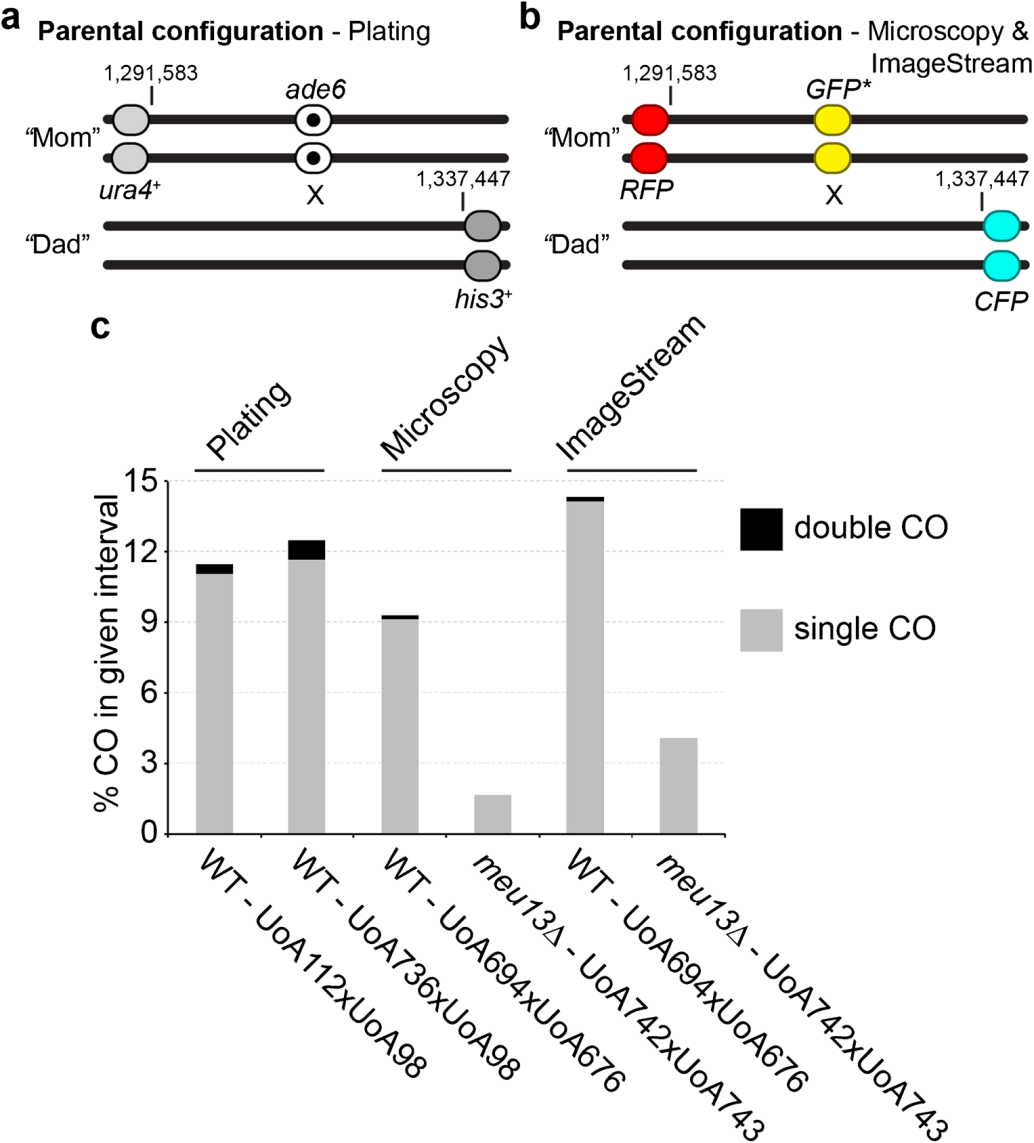
Comparison of genetic intervals generated by nutritional markers and spore-autonomously expressed fluorescent markers. **a** Schematic of genetic recombination assay using nutritional markers and plating of colonies. In UoA112, the *ade6* marker is a point mutation *(ade6-704*) without hotspot activity; in UoA736, it is a partial deletion of *ade6* by integrating a *natMX6* cassette (*ade6-3′*Δ). In both instances, *ade6* is at its endogenous locus on chromosome 3, and the position for the coding sequence is 1,316,337-1,317,995. The flanking markers *ura4*^+^ and *his3*^+^ are the artificially introduced markers (aim) and *his3*^+^-*aim*, which have been previously described (Osman et al. 2003); *ura4*^+^-*aim2* is integrated on chromosome 3 at position 1,291,583, and *his3*^+^-*aim* at position 1,337,447. **b** Schematic of spore-autonomously expressed fluorophore recombination assay (see also Fig. 3a), the *RFP* gene is at the same position as *ura4*^+^-*aim2* in **a**, the *CFP* gene at the same position as *his3*^+^-*aim* in **a**, and the GFP* gene is inserted downstream of *ade6*^+^. **c** Results from recombination assays in **a** and **b**: crossover (CO) recombinant frequencies were determined in wild-type (WT) crosses by random spore analysis for the plating assay (**a**), using data from *n* = 3 independent crosses with 160 progeny each. CO recombinant frequencies were determined in WT and *meu13*Δ crosses either by counting manually on an epi-fluorescence microscope (UoA694 × UoA676 *n* = 356 asci, UoA742 × UoA743 *n* = 305 asci) or by high-throughput single cell assessment on an imaging flow cytometer (ImageStream) (UoA694 × UoA676 *n* = 916 asci, UoA742 × UoA743 *n* = 370 asci). Please note that ImageStream can only identify one out of two double CO classes

Because the fluorophore markers were inserted at the same positions as the nutritional markers of an established recombination assay (Figs. 4a, b and S1a, b) (Osman et al. 2003; Lorenz et al. 2012, 2014), we could directly compare the outcomes of the different assays assessed by various methods. We used two slightly different recombination assays utilizing nutritional markers: one contained a point mutation at *ade6* (*ade6-704*, a T645A substitution mutation; Park et al. 2007), the other one a dominant drug resistance marker inserted at the 3′ end of *ade6* creating a partial deletion (*ade6-3′*Δ::*natMX6*). We used the latter to test whether a drastically different recombination frequency is caused by introducing a heterologous piece of DNA into the genetic interval. The *natMX6* cassette is ~ 1.25 kb in size and removes 848 bp of genomic DNA at *ade6* (429 bp of which are *ade6* coding sequence); in comparison, the spore-autonomously expressed GFP* cassette is ~ 2.1 kb in size and inserted just downstream of the *ade6* open reading frame (removing 73 bp just downstream of the 3′-untranslated region of *ade6*).

Despite all these differences between the markers, the recombination frequencies within the genetic intervals were remarkably similar (Figs. 4c and S1c). The genetic intervals with the nutritional markers produced 11.88% (*ade6-704*) and 13.33% (*ade6-3′*Δ) COs, respectively (Figs. 4c, Table S4). The interval with the fluorophore markers measured 9.41% COs on the epi-fluorescence microscope and 14.57% COs on the imaging flow cytometer (Fig. 4c, Table S5). The results were comparable, when the *ade6*- or GFP*-markers were initially linked with *his3*^+^-*aim* or *CFP*, respectively (10.63% CO for *ade6-704*, 8.33% CO for *ade6-3′*Δ, 7.68% CO for fluorophore markers evaluated by epi-fluorescence microscopy; Fig. S1, Tables S4 and S5). In all types of assays, we could also detect a few rare double CO events (Figs. 4 and S1, Tables S4 and S5). Because asci can be evaluated as an ordered tetrad in the fluorophore-based assay (Figs. 2b and 3a), information about the involvement of two, three, or all four chromatids in the double CO can be extracted. Within the four double CO events over the two slightly different genetic intervals evaluated on the epi-fluorescence microscope (Figs. 4b and S1b), examples for participation of two, three, or four chromatids could be found (Figs. S2 and S3). The observed frequency of double CO in any of the genetic assays is equal with or slightly higher than expected from the frequency in neighboring intervals (Tables S4 and S5), in line with *Sz. pombe* not displaying CO interference (Munz 1994).

In a *meu13* mutant meiotic intra- and intergenic recombination is strongly decreased (Nabeshima et al. 2001). When running the fluorophore-based assay in a *meu13*Δ background, as expected, a 3.6- to 5.7-fold reduction in CO formation could be observed (Fig. 4c). No double COs were detected in the *meu13*Δ crosses. This demonstrates that in *Sz. pombe* a genetic interval consisting of spore-autonomously expressed fluorescent markers behaves very similarly to a genetic interval built from nutritional markers.

## Conclusion

Here, we established assays employing spore-autonomously expressed fluorescent proteins to determine meiotic chromosome mis-segregation and meiotic recombination frequencies in the fission yeast, *Sz. pombe*. We generated a series of plasmids containing selectable markers in addition to the spore-specific fluorophores (Fig. 1, Table S1); this makes the whole system portable enabling the creation of genetic intervals at virtually any position within the *Sz. pombe* genome.

Ectopic spore-autonomous promoters from *Sz. japonicus* work in *Sz. pombe*; this raises the possibility that expression from this type of regulatory elements is conserved, and could be used to develop a similar system in *Sz. japonicus*. This is of interest, because *Sz. japonicus* produces 8-spored asci (an additional mitosis following the two meiotic divisions) (Klar 2013) enabling an even better resolution of genetic events. We validated our system by comparison to an established recombination assay (Osman et al. 2003; Lorenz et al. 2012, 2014) utilizing nutritional markers (Fig. 4), and demonstrated that imaging flow cytometry can be used to run genetic high-throughput screens for recombination phenotypes (Figs. 3 and 4). Due to its portability and advantages over existing assays, our fluorophore-based system represents a novel addition to the ever-growing genetic toolkit for probing the cell biology of fission yeast.

## Electronic supplementary material

ESM 1 (DOCX 704 kb)
