# Supplementary material for: Immediate visualization of recombination events and chromosome segregation defects in fission yeast meiosis
Source: Chromosoma. 2019 Feb 9;128(3):385–96. doi: 10.1007/s00412-019-00691-y (PMC6823302; doi:10.1007/s00412-019-00691-y)
Supplement: Supplementary file 1 — (DOCX 704 kb) [file 412_2019_691_MOESM1_ESM.docx]

Supplementary Materials

**Immediate visualization of recombination events and chromosome segregation defects in fission yeast meiosis**

Dmitriy Li^1,2^, Marianne Roca^1,3^, Raif Yuecel^1,2^ & Alexander Lorenz^1,^*

^1^Institute of Medical Sciences (IMS) and ^2^Iain Fraser Cytometry Centre (IFCC), University of Aberdeen, Foresterhill, Aberdeen AB25 2ZD, UK

^3^Present address: Laboratoire de Biologie du Développement de Villefranche-sur-Mer (LBDV), Sorbonne Université, 06230 Villefranche-sur-Mer, France

*Correspondence should be addressed to

Alexander Lorenz

Phone: +44 1224 437323

E-mail: [a.lorenz@abdn.ac.uk](mailto:a.lorenz@abdn.ac.uk)

ORCID: 0000-0003-1925-3713


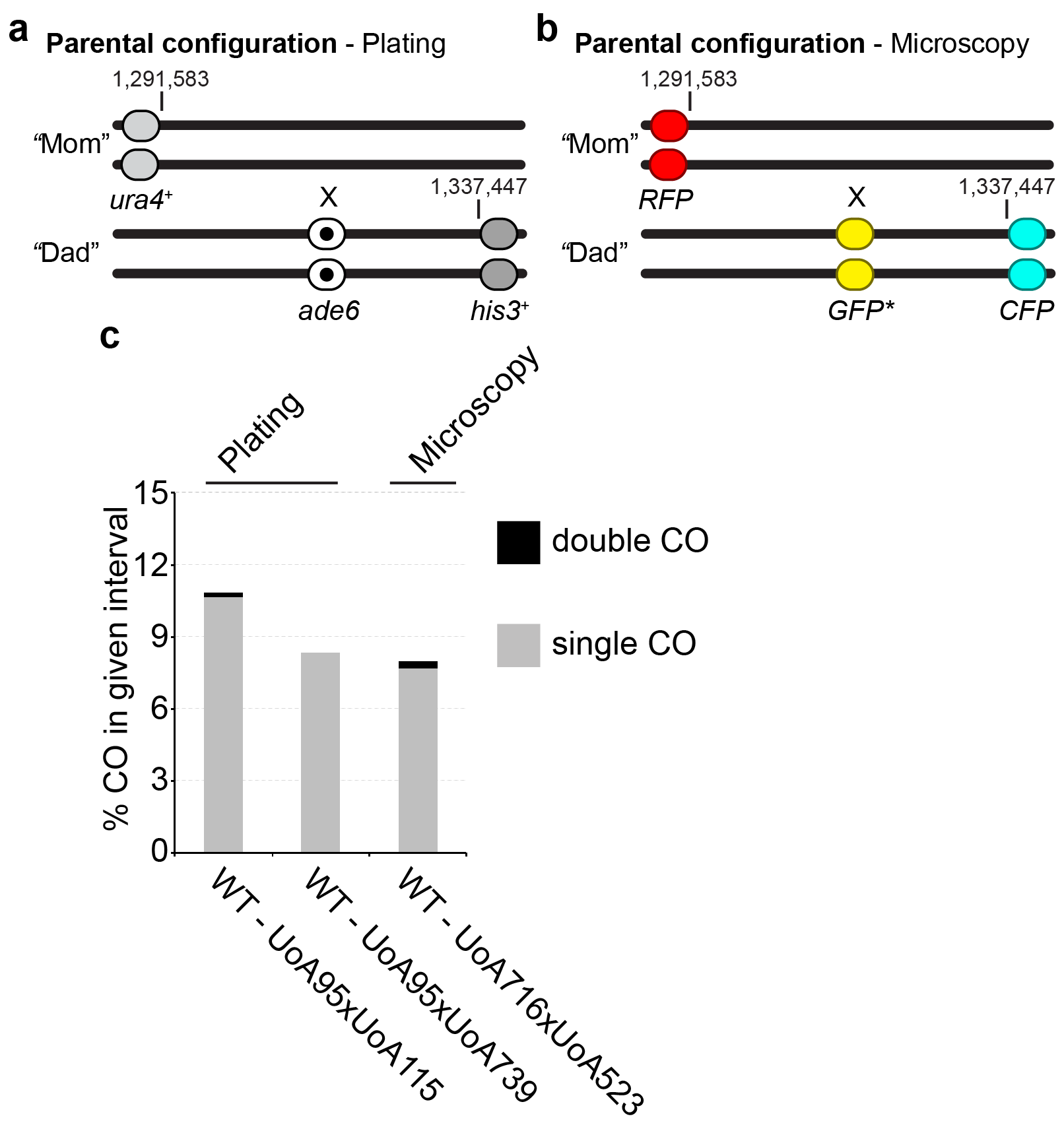


**Figure S1. Comparison of genetic intervals generated by nutritional markers and spore-autonomously expressed fluorescent markers.** (**a**) Schematic of genetic recombination assay using nutritional markers and plating of colonies. In UoA115 the *ade6* marker is a point mutation *(ade6-704*) without hotspot activity, in UoA739 it is a partial deletion of *ade6* by integrating a *natMX6* cassette (*ade6-3’*Δ). In both instances *ade6* is at its endogenous locus on chromosome 3, position for the coding sequence is 1,316,337-1,317,995. The flanking markers *ura4*^+^ and *his3*^+^ are the artificially introduced markers (aim) *ura4*^+^-*aim2* and *his3*^+^-*aim*, which have been previously described (Osman et al. 2003); *ura4*^+^-*aim2* is integrated on chromosome 3 at position 1,291,583, and *his3*^+^-*aim* at position 1,337,447. (**b**) Schematic of spore-autonomously expressed fluorophore recombination assay, the *RFP* gene is at the same position as *ura4*^+^-*aim2* in (a), the *CFP* gene at the same position as *his3*^+^-*aim* in (a), and the *GFP** gene is inserted downstream of *ade6*^+^. (**c**) Results from recombination assays in (a) and (b): crossover (CO) recombinant frequencies were determined in wild-type (WT) crosses by random spore analysis for the plating assay (a), using data from n = 3 independent crosses with 160 progeny each. CO recombinant frequencies were determined in WT and *meu13*Δ crosses either by counting manually on an epi-fluorescence microscope (UoA716×UoA523 n = 508 asci).


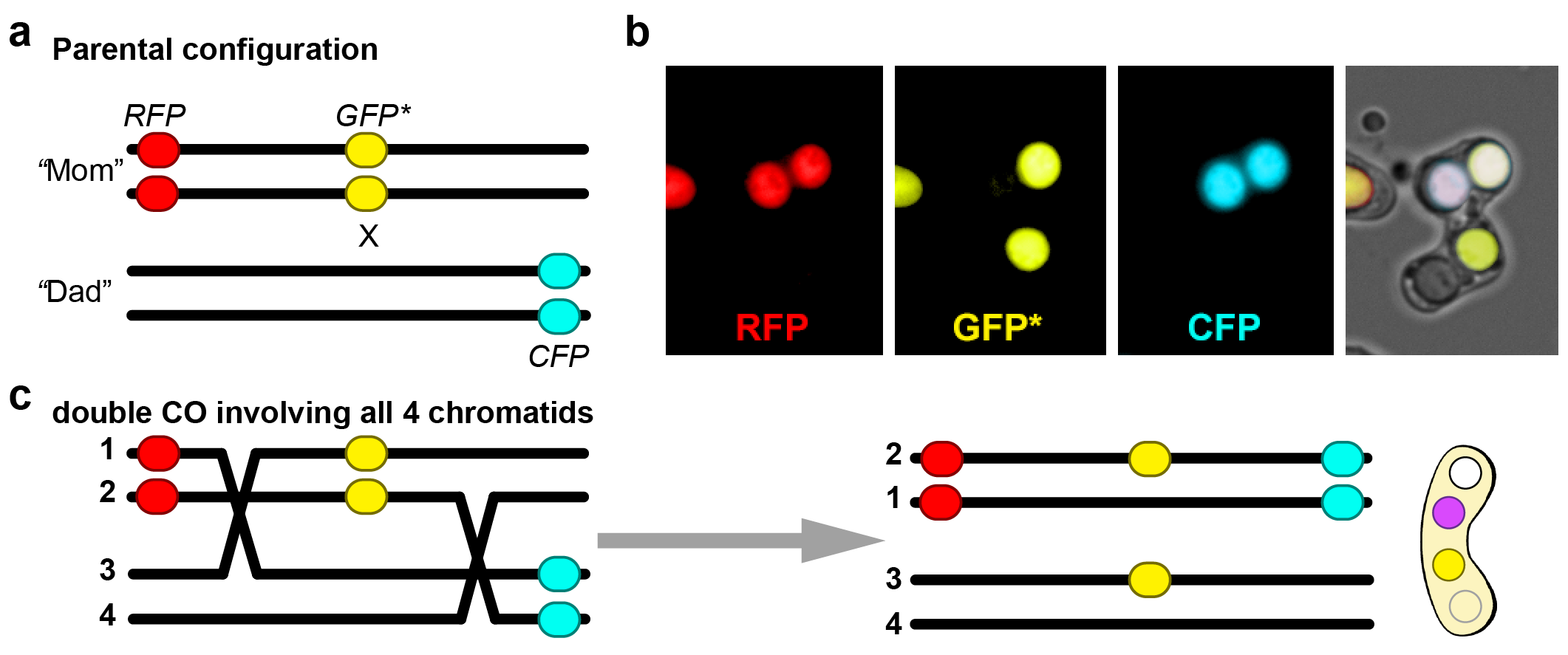


**Figure S2. Double crossover (CO) observed in genetic interval with spore-autonomously expressed fluorescent markers.** (**a**) Schematic of spore-autonomously expressed fluorophore recombination assay for cross UoA694×UoA676 (see also Figs. 3 & 4). (**b**) The only double CO event observed among 356 asci; RFP, GFP*, and CFP fluorescence channels are shown separately and as a merged image. (**c**) Most parsimonious explanation for this ascus phenotype is a double CO involving all 4 chromatids (numbered on the left); possible alternative interpretations would require a third CO.


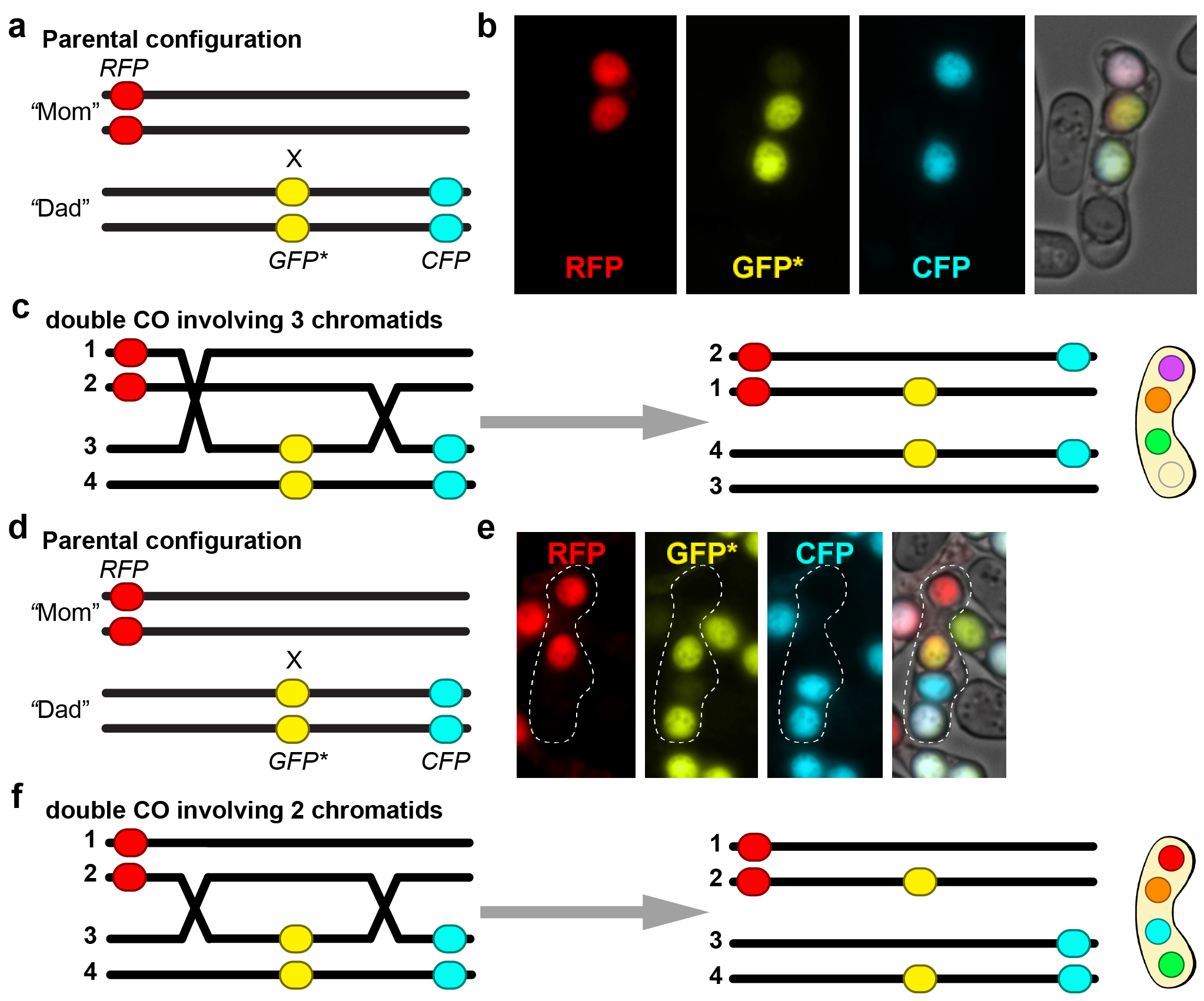


**Figure S3. Double crossover (CO) observed in genetic interval with spore-autonomously expressed fluorescent markers.** (**a**) Schematic of spore-autonomously expressed fluorophore recombination assay for cross UoA716×UoA523 (see also Fig. S1). (**b**) Two double CO events observed among 508 asci; RFP, GFP*, and CFP fluorescence channels are shown separately and as a merged image (one of the two asci is given as example). (**c**) Most parsimonious explanation for this ascus phenotype is a double CO involving 3 chromatids (chromatids are numbered on the left). (**d**) Schematic of spore-autonomously expressed fluorophore recombination assay for cross UoA716×UoA523 (see also Fig. S1). (**e**) One double CO events observed among 508 asci; RFP, GFP*, and CFP fluorescence channels are shown separately and as a merged image. (**f**) Most parsimonious explanation for this ascus phenotype is a double CO involving 2 chromatids only (chromatids are numbered on the left).

**Supplementary Table S1. Plasmid list**

| **Name** | **Relevant Insert/Purpose** | **Ontogeny/Origin** |
| --- | --- | --- |
| pFA6a-*kanMX6* | gene targeting | (Bähler et al. 1998) |
| pALo136/pFA6a* | general cloning vector | re-ligated PCR: oUA2-oUA40 on pFA6a-*kanMX6* after *Bgl*II/*Dpn*I digest |
| pALo145_I | *ura4*^+^ | *ura4*^+^-*Hind*III^a^ fragment into pALo136 |
| pALo146 | *his3*^+^ | *his3*^+^-*Pst*I/*Sal*I^b^ fragment into pALo136 |
| pALo185 | *arg3*^+^ | *arg3*^+^ [PCR (oUA211-212) on pFA6a-*arg3MX4*]^c^ as a *Pvu*II/*Pst*I fragment into pALo136 |
| pALo158 | 3’ end of *ade6* | PCR (oUA65-oUA130) on gDNA (ALP714) as a *Hind*III/*Bam*HI fragment into pALo136 |
| pALo159 | 3’ downstream flanking sequence of *ade6* | PCR (oUA131-oUA132) on gDNA (ALP714) as a *Bgl*II/*Eco*RV fragment into pALo158 |
| pCR2.1-nat | gene targeting | (Sato et al. 2005) |
| pALo169 | *natMX6 –* targeting to *ade6* | *natMX6* from pCR2.1-nat as an *Eco*RI fragment into pALo159 |
| pSK691 | *P_YLK050c(Skud)_-*tdTomato*-T_PGK1(Skud)_* | (Thacker et al. 2011) |
| pSK692 | *P_YLK050c(Sbay)_*-mCerulean-*T_PGK1(Sbay)_* | (Thacker et al. 2011) |
| pSK726 | *P_YLK050c(Smik)_*-GFP*-*T_PGK1(Smik)_* | (Thacker et al. 2011) |
| pDUAL | targeting to *leu1-32* | (Matsuyama et al. 2004) |
| pALo137 | *tdTomato-T_PGK1(Skud)_* (targeting to *leu1-32*) | PCR (oUA67-oUA68) on pSK691 as a *Bss*HII/*Spe*I fragment into pDUAL |
| pALo138 | mCerulean-*T_PGK1(Sbay)_* (targeting to *leu1-32*) | PCR (oUA67-oUA69) on pSK692 as a *Bss*HII/*Spe*I fragment into pDUAL |
| pALo139 | *P_eis1_-tdTomato-T_PGK1(Skud)_* (targeting to *leu1-32*) | PCR (oUA70-oUA71) on gDNA (UoA431) as a *Sph*I/*Bss*HII fragment into pALo137 |
| pALo140 | *P_eng2_-*tdTomato*-T_PGK1(Skud)_* (targeting to *leu1-32*) | PCR (oUA72-oUA73) on gDNA (UoA431) as a *Sph*I/*Bss*HII fragment into pALo137 |
| pALo141 | *P_agn2_-* mCerulean-*T_PGK1(Sbay)_* (targeting to *leu1-32*) | PCR (oUA74-oUA75) on gDNA (UoA431) as a *Sph*I/*Bss*HII fragment into pALo138 |
| pALo142 | *P_mde10_-* mCerulean-*T_PGK1(Sbay)_* (targeting to *leu1-32*) | PCR (oUA76-oUA77) on gDNA (UoA431) as a *Pst*I/*Bss*HII fragment into pALo138 |
| pALo175 | *P_pil2_-* mCerulean-*T_PGK1(Sbay)_* (targeting to *leu1-32*) | PCR (oUA193-oUA194) on gDNA (ALP1596) as a *Pst*I/*Bss*HII fragment into pALo138 |
| pALo148 | *P_SJAG_04227_-*tdTomato*-T_PGK1(Skud)_* (*ura4*^+^-marked, not targeting) | PCR (oUA91-oUA92) on gDNA (yFS275) and PCR (oUA93-oUA94) on pALo137 into pALo145_I after *Pst*I/*Spe*I digest (In-fusion cloning) |
| pALo181 | *P_SPOG_00147_-* tdTomato*-T_PGK1(Skud)_* (*his3*^+^-marked, not targeting) | PCR (oUA205-oUA206) on gDNA (FY21620) and PCR (oUA93-oUA94) on pALo137 into pALo146 after *Sal*I/*Spe*I digest (NEBuilder assembly) |
| pALo182 | *P_SPOG_00147_-* mCerulean-*T_PGK1(Sbay)_* (*his3*^+^-marked, not targeting) | PCR (oUA205-oUA206) on gDNA (FY21620) and PCR (oUA93-oUA98) on pALo142 into pALo146 after *Sal*I/*Spe*I digest (NEBuilder assembly) |
| pALo168 | *P_SPOG_00147_-* mCerulean-*T_PGK1(Sbay)_* (*his3*^+^-marked, targeting to *his3*^+^-aim) | PCR (oUA189-oUA190) & PCR (oUA191-oUA192) on gDNA (MCW1196), insert & vector of pALo182 after *Not*I digest (NEBuilder assembly) |
| pALo179 | *P_SOCG_04642_-* GFP*-*T_PGK1(Smik)_* (targeting to *ade6*) | PCR (oUA201-oUA202) on gDNA (yFS286) and PCR (oUA204-oUA138) on pSK726 into pALo159 after *Bam*HI-*Bgl*II digest (NEBuilder assembly) |
| pALo186 | *P_SOCG_04642_-* GFP*-*T_PGK1(Smik)_* (*arg3*^+^-marked, not targeting) | PCR (oUA264-oUA265) on pALo179 into pALo185 after *Bam*HI digest (NEBuilder assembly) |
| pALo196 | *P_SPOG_00147_-* tdTomato*-T_PGK1(Skud)_* (targeting to *CEN1*) | PCR (oUA230-oUA231) & PCR (oUA232-oUA233) on gDNA (ALP714) into pALo181 after *Pvu*II digest (NEBuilder assembly) |
| pALo197 | *P_SPOG_00147_-* mCerulean-*T_PGK1(Sbay)_* (targeting to *CEN1*) | PCR (oUA230-oUA231) & PCR (oUA232-oUA233) on gDNA (ALP714) into pALo182 after *Pvu*II digest (NEBuilder assembly) |

Unless otherwise stated, plasmid construction was performed via standard T4 DNA ligase-based cloning of restriction endonuclease-digested fragments.

For sequences of pALo-constructs see <https://figshare.com/s/8b8aec968952b862523d>.

^a^The *ura4*^+^-*Hind*III fragment (1,764bp) is a functional Ura^+^ marker (Grimm et al. 1988).

^b^The *his3*^+^-*Pst*I/*Sal*I fragment (2,069bp) is a functional His^+^ marker (Osman et al. 2000).

^c^The *arg3*^+^-*Pvu*II/*Pst*I fragment (1,905bp) is a functional Arg^+^ marker (Waddell and Jenkins 1995; Lorenz 2015).

**Supplementary Table S2. List of Oligonucleotides**

| **Name** | **Sequence (5’ – 3’)** |
| --- | --- |
| oUA2 | GGGACGAGGCAAGCTAAAC |
| oUA40 | AATTAAagatctGAGCTCGAATTCATCGATG |
| oUA65 | ggtgcgcacttttgaagg |
| oUA67 | aattaaGCGCGCatggtgagcaagggcgagg |
| oUA68 | GAATCATCTTACTAGTggatcc |
| oUA69 | aattaaACTAGTgcttggagcagggaggatac |
| oUA70 | aattaaGCATGCGTAGGTACGTTAGCTGG |
| oUA71 | AATTAAgcgcgcTTCAAAATAATATTAGAGTACTGTGATGC |
| oUA72 | aattaaGCATGCGCACTCTCACTCTTTCCG |
| oUA73 | aattaagcgcgcTATGTCGTCTGACTATGAATTGAG |
| oUA74 | GCTAAGAAAAAGCATGCTCG |
| oUA75 | aattaagcgcgcTAAGTGATATTTAAGTTGGTTAGTGG |
| oUA76 | aattaactgcaGGTAAGCCATGCTTAACG |
| oUA77 | aattaagcgcgcAGTATATCATATATTCTTTTTTAAAAAGAGC |
| oUA91 | atcacAAGCTTCGTACGCGCAGAGCGTACAAAATTGC |
| oUA92 | gcccttgctcaccatGGTATGTGTAGTGAATTGAGTAG |
| oUA93 | atggtgagcaagggcgag |
| oUA94 | GGCCGCATAGGCCACTAGccagcttggagcaggaag |
| oUA98 | GGCCGCATAGGCCACTAGTgcttggagcaggg |
| oUA101 | AGCTACAAATCCCACTGG |
| oUA102 | GTGATATTGACGAAACTTTTTG |
| oUA113 | TGGAAATatccgatataagaatattattcataaagagaggtacttaatgtagacggaaaacaccacttattagccgatgcttagctacaaatcccactgg |
| oUA114 | GCTGACAACTTCAAATCCATATTACTTTCGCCTCGCTCTGCTTAGCAGAACCTTTGTTTGAATTTCTACTCGCctagccagcttggagcagg |
| oUA130 | aattaaggatcccaaaacaaaaagcaagc |
| oUA131 | aattaaAGATCTCCCCCCGAATAATGTGCTGC |
| oUA132 | AATTAAgatatcGCCAAACATAATGCGGTCCG |
| oUA138 | ACATTATTCGGGGGGAGATCTgtgggatgagcttggagcag |
| oUA142 | ccaacttctcagtttgaagc |
| oUA143 | CCAAACATAATGCGGTCC |
| oUA189 | CGATTTAGGTGACACTATAGAACGCCcgggCGTCTCGTGAATTGTACG |
| oUA190 | CGTACGAAGCTTCAGCTGGCGAAACATGAGTTATTCACAATTGG |
| oUA191 | ccaagcACTAGTGGCCTATGCAATATATGGAGCATTAAACACTATTTAAATTTGC |
| oUA192 | CTATAGGGAGACCGGCAGATCCGCccgggCTGAGAAAACAAGCCGTGC |
| oUA193 | GCCACCAGCTCATTCTGC |
| oUA194 | AATTAAgcgcgCCATCATCTTGCTAATTACTTGATAAACG |
| oUA201 | gcttgctttttgttttgggatccGCTTGATAAGGTCTCTTCTCGC |
| oUA202 | GTTCCTCGCCCTTGCTCACCatCTTGAAAATGATTGATCACTTAACTGCTG |
| oUA204 | GGTGAGCAAGGGCGAGGAAC |
| oUA205 | ccaatcaagcttatcgataccGTCGACGCGCTTAGTTTTGTATTGCCG |
| oUA206 | cgcccttgctcaccatCTTAAAAATGATTGATCACTTAAGCGC |
| oUA211 | AATTAAcagctgACGTACTAGCTTGTTTGC |
| oUA212 | AATTAActgcaGGAAGACAAGAAAAAGCC |
| oUA230 | CCAACAgggcccGGATTTGGCAATCTCTTTGC |
| oUA231 | TGCAGCGTACGAAGCTTCAGCCAAACACCAAGTAGACACG |
| oUA232 | CTATAGAACGCGGCCGCCAGCTGTTTGGAAAATCGTACCTAG |
| oUA233 | GCCAAATCCgggcccTGTTGGTCATCGGTTCG |
| oUA264 | GCCTTAATTAACCCGGGGATCCGCTTGATAAGGTC |
| oUA265 | CTTCCTGCAGGTCGACGGATCTgtgggatgagcttg |

**Supplementary Table S3. Yeast strain list** (in order of appearance)

| **Strain** | **Relevant genotype** | **Origin** |
| --- | --- | --- |
| UoA727 | *h^-smt0^ CEN1*::*his3*^+^*-P_SPOG_00147_-mCerulean arg3-D4 his3-D1 leu1-32 ura4-D18* | this study |
| UoA726 | *h^+S^ CEN1*::*his3*^+^*-P_SPOG_00147_-tdTomato arg3-D4 his3-D1 leu1-32 ura4-D18* | this study |
| UoA694 | *h^-smt0^ ura4*^+^*-aim2-P_SJAG_04227_-tdTomato-T_PGK1(Skud)_ ade6*^+^::*P_SOCG_04642_- GFP**-*T_PGK1(Smik)_ arg3-D4 his3-D1 leu1-32 ura4-D18* | this study |
| UoA752 | *h^+S^ meu13*Δ*-43*::*natMX4 CEN1*::*his3*^+^*-P_SPOG_00147_-tdTomato arg3-D4 his3-D1 leu1-32 ura4-D18* | this study |
| UoA755 | *h^-smt0^ meu13*Δ*-43*::*natMX4 CEN1*::*his3*^+^*-P_SPOG_00147_-mCerulean arg3-D4 his3-D1 leu1-32 ura4-D18* | this study |
| UoA756 | *h^+S^ meu13*Δ*-43*::*natMX4 sgo1*Δ::*hphMX4 CEN1*::*his3*^+^*-P_SPOG_00147_-tdTomato arg3-D4 his3-D1 leu1-32 ura4-D18* | this study |
| UoA759 | *h^-smt0^ meu13*Δ*-43*::*natMX4 sgo1*Δ::*hphMX4 CEN1*::*his3*^+^*-P_SPOG_00147_-mCerulean arg3-D4 his3-D1 leu1-32 ura4-D18* | this study |
| UoA760 | *h^+S^ rec12*Δ*-169*::*3HA6His-kanMX6 sgo1*Δ::*hphMX4 CEN1*::*his3*^+^*-P_SPOG_00147_-tdTomato arg3-D4 his3-D1 leu1-32 ura4-D18* | this study |
| UoA763 | *h^-smt0^ rec12*Δ*-169*::*3HA6His-kanMX6 sgo1*Δ::*hphMX4 CEN1*::*his3*^+^*-P_SPOG_00147_-mCerulean arg3-D4 his3-D1 leu1-32 ura4-D18* | this study |
| UoA676 | *h^+S^ his3*^+^*-aim-P_SPOG_00147_-mCerulean*-*T_PGK1(Sbay)_ arg3-D4 his3-D1 leu1-32 ura4-D18* | this study |
| UoA112 | *h^+S^ ade6-704 ura4^+^-aim2 his3-D1 leu1-32 ura4-D18* | this study |
| UoA736 | *h^+S^ ade6-3’*Δ::*natMX6 ura4^+^-aim2 his3-D1 leu1-32 ura4-D18* | this study |
| UoA98 | *h^-smt0^ his3^+^-aim arg3-D4 his3-D1 ura4-D18* | this study |
| UoA742 | *h^+S^ meu13*Δ*-22*::*hphMX4 his3*^+^*-aim-P_SPOG_00147_-mCerulean*-*T_PGK1(Sbay)_ arg3-D4 his3-D1 leu1-32 ura4-D18* | this study |
| UoA743 | *h^-smt0^ meu13*Δ*-22*::*hphMX4 ura4*^+^*-aim2-P_SJAG_04227_-tdTomato-T_PGK1(Skud)_ ade6*^+^::*P_SOCG_04642_- GFP**-*T_PGK1(Smik)_ arg3-D4 his3-D1 leu1-32 ura4-D18* | this study |
| UoA716 | *h^+S^ his3*^+^*-aim-P_SPOG_00147_-mCerulean*-*T_PGK1(Sbay)_ ade6*^+^::*P_SOCG_04642_-GFP**-*T_PGK1(Smik)_ arg3-D4 his3-D1 leu1-32 ura4-D18* | this study |
| UoA570 | *h^+S^ ade6-3’*Δ::*natMX6 arg3-D4 his3-D1 leu1-32 ura4-D18* | this study |
| UoA523^a^ | *h^-smt0^ ura4*^+^*-aim2-P_SJAG_04227_-tdTomato*-*T_PGK1(Skud)_ arg3-D4 his3-D1 leu1-32 ura4-D18* | this study |
| UoA666 | *h^+S^ ade6*^+^::*P_SOCG_04642_-GFP**-*T_PGK1(Smik)_ arg3-D4 his3-D1 leu1-32 ura4-D18* | this study |
| UoA115 | *h^-smt0^ ade6-704 his3^+^-aim arg3-D4 his3-D1 ura4-D18* | this study |
| UoA739 | *h^-smt0^ ade6-3’*Δ::*natMX6 his3^+^-aim arg3-D4 his3-D1 ura4-D18* | this study |
| UoA95 | *h^+S^ ura4^+^-aim2 his3-D1 leu1-32 ura4-D18* | this study |
| ALP729 | *h^+S^ arg3-D4 his3-D1 leu1-32 ura4-D18* | (Lorenz et al. 2012) |
| FO652 | *h^-smt0^ arg3-D4 his3-D1 leu1-32 ura4-D18* | lab strain |
| UoA581 | *h^-^ meu13*Δ*-22*::*hphMX4 ade6-M210 leu1-32 ura4-D18* | (Lorenz 2015) |
| UoA585 | *h^+S^ meu13*Δ*-22*::*hphMX4 arg3-D4 his3-D1 leu1-32 ura4-D18* | this study |
| UoA723 | *h^+S^ meu13*Δ*-43*::*natMX4 arg3-D4 his3-D1 leu1-32 ura4-D18* | this study |
| JG17888 | *h^-^ sgo1*Δ::*hphMX4 ade6-M210 leu1-32 ura4-D18* | (Gregan et al. 2005) |
| GP3717 | *h^+^ rec12*Δ*-169*::*3HA6His-kanMX6 ade6-M216* | (Davis and Smith 2003) |
| ALP714 | *h^+S^* | (Lorenz et al. 2012) |
| UoA431 | *h^+N^ ade6-469* | this study |
| ALP1596 | *h^-smt0^ ade7-152 his3-D1 leu1-32 ura4-D18* | (Lorenz et al. 2014) |
| yFS275^b^ | *Schizosaccharomyces japonicus, h^90^* | (Rhind et al. 2011) |
| FY21620^c^ | *Schizosaccharomyces cryophilus* | (Helston et al. 2010) |
| MCW1196 | *h^+S^ his3^+^-aim ade6-469 his3-D1 leu1-32 ura4-D18* | (Osman et al. 2003) |
| yFS286^d^ | *Schizosaccharomyces octosporus, h^90^* | (Rhind et al. 2011) |

Unless otherwise noted, strains listed belong to *Schizosaccharomyces pombe*.

^a^*ura4*^+^*-aim2-P_SJAG_04227_-tdTomato*-*T_PGK1(Skud)_* was transformed into strain FO652 as a PCR product (oUA113-oUA114) with ~80nt homologous overhangs as described by Bähler et al. (1998).

^b^provided as FY16936 by the National BioResource Project (NBRP) of the MEXT, Japan.

^c^provided by the National BioResource Project (NBRP) of the MEXT, Japan.

^d^provided as FY16937 by the National BioResource Project (NBRP) of the MEXT, Japan.

**Supplementary Table S4. Recombination frequency in the interval *ura4*^+^-*aim2* – *ade6-704*/*ade6-3’*Δ – *his3*^+^-*aim* by random spore analysis**

| **cross** | **% spore viability [±S.D.]** | **% total CO [±S.D.]** | **% CO *ura4*-*ade6* [±S.D.]** | **% CO *ade6-his3* [±S.D.]** | **% double CO [±S.D.]** | **% double CO (exp.)** |
| --- | --- | --- | --- | --- | --- | --- |
| UoA112×UoA98 | 74.7 [±7.84] | 11.88 [±4.1] | 6.88 [±3.13] | 5.0 [±1.08] | 0.42 [±0.72] | 0.34 |
| UoA736×UoA98 | 74.6 [±5.59] | 13.33 [±1.3] | 5.83 [±0.96] | 7.5 [±1.25] | 0.83 [±0.36] | 0.44 |
| UoA95×UoA115 | 87.6 [±2.91] | 11.04 [±2.95] | 6.67 [±2.95] | 4.38 [±0.0] | 0.21 [±0.36] | 0.74 |
| UoA95×UoA739 | 76.7 [±15.1] | 8.33 [±3.08] | 6.04 [±1.8] | 2.29 [±1.3] | 0.0 | 0.14 |

Crossover (CO) frequency expressed as (number of recombinant progeny)/(total viable colonies) in %.

**Supplementary Table S5. Recombination frequency in the interval *RFP* – *GFP** – *CFP***

| **cross** | **% total CO** | **% CO *RFP*-*GFP**** | **% CO *GFP*-CFP*** | **% double CO** | **% double CO (exp.)** |
| --- | --- | --- | --- | --- | --- |
| UoA716×UoA523 | 8.29 | 1.97 | 6.3 | 0.295 | 0.124 |
| UoA694×UoA676 | 9.41 | 1.55 | 7.87 | 0.14 | 0.121 |
| UoA742×UoA743 | 1.63 | 0.33 | 1.31 | 0.00 | 0.0043 |

Crossover (CO) frequency expressed as (2× number of recombinant asci)/(4× total number of asci) in %.

**Supplementary References**

Bähler J, Wu JQ, Longtine MS, et al (1998) Heterologous modules for efficient and versatile PCR-based gene targeting in *Schizosaccharomyces pombe*. Yeast 14:943–51. doi: 10.1002/(SICI)1097-0061(199807)14:10<943::AID-YEA292>3.0.CO;2-Y

Davis L, Smith GR (2003) Nonrandom homolog segregation at meiosis I in *Schizosaccharomyces pombe* mutants lacking recombination. Genetics 163:857–74.

Gregan J, Rabitsch PK, Sakem B, et al (2005) Novel genes required for meiotic chromosome segregation are identified by a high-throughput knockout screen in fission yeast. Curr Biol 15:1663–9. doi: 10.1016/j.cub.2005.07.059

Grimm C, Kohli J, Murray J, Maundrell K (1988) Genetic engineering of *Schizosaccharomyces pombe*: a system for gene disruption and replacement using the *ura4* gene as a selectable marker. Mol Gen Genet 215:81–6. doi: doi.org/10.1007/BF00331307

Helston RM, Box JA, Tang W, Baumann P (2010) *Schizosaccharomyces cryophilus* sp. nov., a new species of fission yeast. FEMS Yeast Res 10:779–86. doi: 10.1111/j.1567-1364.2010.00657.x

Lorenz A (2015) New cassettes for single-step drug resistance and prototrophic marker switching in fission yeast. Yeast 32:703–710. doi: 10.1002/yea.3097

Lorenz A, Mehats A, Osman F, Whitby MC (2014) Rad51/Dmc1 paralogs and mediators oppose DNA helicases to limit hybrid DNA formation and promote crossovers during meiotic recombination. Nucleic Acids Res 42:13723–13735. doi: 10.1093/nar/gku1219

Lorenz A, Osman F, Sun W, et al (2012) The fission yeast FANCM ortholog directs non-crossover recombination during meiosis. Science 336:1585–8. doi: 10.1126/science.1220111

Matsuyama A, Shirai A, Yashiroda Y, et al (2004) pDUAL, a multipurpose, multicopy vector capable of chromosomal integration in fission yeast. Yeast 21:1289–305. doi: 10.1002/yea.1181

Osman F, Adriance M, McCready S (2000) The genetic control of spontaneous and UV-induced mitotic intrachromosomal recombination in the fission yeast *Schizosaccharomyces pombe*. Curr Genet 38:113–25.

Osman F, Dixon J, Doe CL, Whitby MC (2003) Generating crossovers by resolution of nicked Holliday junctions: a role for Mus81-Eme1 in meiosis. Mol Cell 12:761–74. doi: 10.1016/S1097-2765(03)00343-5

Rhind N, Chen Z, Yassour M, et al (2011) Comparative functional genomics of the fission yeasts. Science 332:930–6. doi: 10.1126/science.1203357

Sato M, Dhut S, Toda T (2005) New drug-resistant cassettes for gene disruption and epitope tagging in *Schizosaccharomyces pombe*. Yeast 22:583–91. doi: 10.1002/yea.1233

Thacker D, Lam I, Knop M, Keeney S (2011) Exploiting spore-autonomous fluorescent protein expression to quantify meiotic chromosome behaviors in *Saccharomyces cerevisiae*. Genetics 189:423–39. doi: 10.1534/genetics.111.131326

Waddell S, Jenkins JR (1995) arg3+, a new selection marker system for *Schizosaccharomyces pombe*: application of *ura4*+ as a removable integration marker. Nucleic Acids Res 23:1836–7.
